# Comorbidity of Histamine Intolerance and Polyvalent Allergy: A Case Report and Literature Review

**DOI:** 10.3390/healthcare13020094

**Published:** 2025-01-07

**Authors:** Oksana Wojas, Edyta Krzych-Fałta, Paweł Pihowicz, Paulina Żybul, Anna Szylling, Bolesław Samoliński

**Affiliations:** 1 Department of Prevention of Environmental Hazards Allergology and Immunology, Medical University of Warsaw, 02-097 Warsaw, Poland; oksana.wojas@wum.edu.pl (O.W.); anna@szylling.pl (A.S.); boleslaw.samolinski@wum.edu.pl (B.S.); 2 Department of Basic Nursing, Medical University of Warsaw, 01-445 Warsaw, Poland; 3 Department of Pathology, Medical University of Warsaw, Pawińskiego 3B, 02-106 Warsaw, Poland; pawel.pihowicz@wum.edu.pl; 4 Department of Gastroenterology and Internal Medicine, Medical University of Warsaw, 02-097 Warsaw, Poland; paulina.zybul@uckwum.pl

**Keywords:** histamine, histamine intolerance, diamine oxidase, multimorbidity, food allergy

## Abstract

**Background/Objectives:** Histamine intolerance is becoming a critical medical problem across numerous clinical specialties, due to the absence of a standardized diagnostic and therapeutic strategy to manage patients with a suspicion of or diagnosis of this condition. Histamine intolerance is a type of non-immune food hypersensitivity, characterized by heterogenous etiologies and a very broad range of symptoms. The condition is the result of an imbalance between the amount of histamine accumulated within the body and the body’s systemic ability to degrade it. In regard to the diagnostics of histamine intolerance, the need to preliminarily exclude other potential conditions associated with increased histamine levels in the blood has been highlighted. The co-occurrence of allergies and histamine intolerance is not uncommon, and the similarity of the clinical manifestations can lead to diagnostic, as well as therapeutic, difficulties. This paper details the diagnostic and clinical workflow for a patient with histamine intolerance and polyvalent allergy comorbidity, with the aim being to help outline a protocol that may be helpful to clinicians managing patients with histamine intolerance. **Case Presentation**: This article presents the case of a 30-year-old patient with a polyvalent allergy and multimorbidity (allergic rhinitis, asthma, a food allergy, and eosinophilic esophagitis), with comorbid histamine intolerance. Due to the violent and severe symptoms, including facial erythema, urticaria, pruritus, abdominal pain, and tachycardia, experienced after meals, the patient received intramuscular epinephrine injections three times a week. The diagnostic protocol and the course of therapeutic management are presented. **Conclusions**: The diagnosis of histamine intolerance is difficult due to the high variability and heterogeneity of clinical symptoms in individual patients. Many studies on the issue recommend ruling out an allergic background in terms of the complaint. However, the possibility of the symptoms of an IgE-dependent allergy overlapping with those of histamine intolerance should be taken into account in every case. This is particularly important in patients presenting with an atypical and severe course of allergic diseases. The clinical case presented herein may be helpful for the daily practice of allergologists and physicians with other specialties, as an example of multimorbidity with both allergic and non-allergic backgrounds.

## 1. Introduction

Histamine intolerance (HIT) has become an extremely important medical problem in regard to the practice of physicians in numerous specialties. Interest in the problem has increased significantly in recent years, as reflected by the numerous scientific publications on the matter; however, physicians continue to face challenges related to the absence of standardized diagnostic protocols, as well as therapeutic and management strategies, for use in patients with the suspicion or diagnosis of this condition [1].

Histamine is a biogenic amine formed during an endogenous biosynthetic process from the amino acid, histidine. It is involved in numerous physiological processes. Histamine exerts its effects via four receptors (H1, H2, H3, and H4). Endogenous histamine is stored mainly within mast cells and basophils. Both endogenous and exogenous histamine is eliminated by two metabolic pathways: methylation, by histamine N-methyltransferase, and oxidative degradation, by diamine oxidase (DAO) [2,3]. In 2011, the European Food Safety Authority (EFSA) published a scientific report on the content of biogenic amines in food marketed within the EU and the risk to consumers’ health. Of these biogenic amines, histamine has been found to have the greatest toxic potential [4]. The first report on the negative effects of histamine, consumed as a result of being ingested with food, on the human body dates back to the 1940s. In 1947, Legroux et al. described the onset of symptoms of mass poisoning following the consumption of tuna meat, which was linked to significantly elevated histamine concentrations in the flesh of these fishes [5]. Histamine poisoning is associated with acute ingestion of an excessive dose of histamine (>500 mg) that is greater than a healthy individual’s ability to break it down, although cases of poisoning symptoms presenting after the ingestion of 100 mg and severe poisoning after the ingestion of 1000 mg have also been reported [1,2,3]. Histamine poisoning most often occurs after the consumption of improperly stored fish from the mackerel family (*Scombridae*), such as mackerel or tuna. The meat from this species of fish is naturally rich in free L-histidine [6]. Since histamine poisoning can also be caused by other foods that are high in histamine, including cheeses, the World Health Organization (WHO) has recommended the use of the more precise term, histamine intoxication [7]. In the 1980s, the first cases of adverse reactions following the ingestion of even small amounts of histamine, considered harmless to healthy people, were observed and the phenomenon was referred to as histamine hypersensitivity (intolerance) [1,8].

Histamine intolerance is a type of non-immune food hypersensitivity, characterized by heterogeneity in terms of the causes and a very broad range of symptoms. The condition is the result of an imbalance between the amount of histamine accumulated within the body and the body’s systemic ability to degrade it [1,8]. Most commonly, it is also accompanied by reduced activity of the intestinal enzyme, diamine oxidase (DAO). DAO was first identified in 1929 by Charles H. Best, during the process of autolysis of lung tissue; at the time, the name histaminase was given to the enzyme due to its observable properties [9]. Many years later, the enzyme was found to have the ability to degrade other diamines as well and, hence, the new name, diamine oxidase, was proposed. DAO is a copper-dependent amine oxidase, encoded by the *AOC1* gene located on chromosome 7 (7q34–36). The enzyme is synthesized mainly within the small intestine, ascending colon, placenta, and kidney. In the gastrointestinal tract, DAO is the most important enzyme responsible for degrading either food-supplied or endogenous histamine [8,10]. The most important of the numerous known causes of DAO deficiency include genetic factors, inflammatory changes, and the use of DAO inhibitors. Reduced DAO activity may result from genetic changes, i.e., single nucleotide polymorphisms within the *AOC1* gene. As of now, 85 different single nucleotide polymorphism (SNP) variants have been identified within the human DAO enzyme-encoding gene, some of which may lead to reduced enzymatic activity. DAO is mainly expressed within the small intestine and, therefore, inflammation of gastrointestinal mucosa may cause a decrease in its synthesis. The most common mutations resulting in reduced DAO activity include rs1049793, rs10156191, rs1049742, and rs2268999 polymorphisms [3]. The most important inflammatory pathologies include Crohn’s disease, ulcerative colitis, irritable bowel syndrome, short bowel syndrome, parasitic infections, celiac disease, and non-celiac gluten sensitivity. Following the ingestion of certain drugs that block DAO or stimulate the release of endogenous histamines, the systemic metabolism of the substance can also be disturbed. Notably, one of the key factors affecting abnormal histamine metabolism is alcohol consumption [1,3,8,9]. Clinical manifestations of histamine intolerance are varied and not always specific; in addition, histamine intolerance may be caused by different pathophysiological mechanisms or a combination of such mechanisms. For this reason, the diagnosis of the disorder poses numerous difficulties. Since the symptoms are not pathognomonic, the probability of their occurrence being associated with the consumption of histamine-rich foods should be ascertained in the first place. In regard to the diagnosis of histamine intolerance, the most important part consists in the exclusion of any other potential conditions associated with an increase in histamine levels in the blood. In regard to a differential diagnosis, the possible existence of an IgE-dependent allergy should be considered and an allergen-specific IgE panel or skin prick tests (SPTs) should be carried out, in regard to the patient, to that end. The possibility of an overlap between an IgE-dependent allergy and histamine intolerance in the same patient should also be kept in mind. There appears to be a pathophysiological connection between allergies and histamine intolerance, characterized by the dysregulation of immune responses and histamine metabolism, leading to overlapping clinical symptoms and the mutual exacerbation of these conditions in some patients [10].

## 2. Case Presentation

In 2021, a 30-year-old man presented at the Allergy Outpatient Clinic with symptoms including facial erythema; an urticaria-like rash on the skin, all over the body; skin itching; watery eyes; watery nasal discharge; abdominal pain; diarrhea; tachycardia; headache; and dizziness. The aforementioned complaints had been occurring for about a year following the ingestion of various meals, albeit the patient was unable to specify precisely the kind of meals responsible for these symptoms. The symptoms usually developed after eating out in restaurants and bars. Suspected products included red wine, blue cheese, tomatoes and tomato preparations, fish, citrus fruit, sauerkraut, and avocado. In addition, after eating fresh carrots, celery, apples, cherries, hazelnuts, and almonds, the patient experienced increased itching in the mouth and swelling of the lips and tongue. The symptoms were usually resolved following a double dose of antihistamines. Most importantly, in the recent period, the patient had resorted to intramuscular epinephrine injections (self-administered using autoinjectors) up to three times a week, in addition to antihistamines. According to the patient’s account, he had called the emergency room several times and was observed within the ED department, albeit he did not provide medical records from these visits. Epinephrine autoinjections were prescribed to the patient by his family physician.

## 3. Medical History, Physical Examination, and Diagnosis

Based on the patient’s history, it was determined that he had experienced symptoms including watery nasal discharge, sneezing, nasal obstruction, watery eyes, and wheezing, in the spring and summer seasons, since early childhood. In addition, he reported experiencing year-round symptoms in the form of persistent nasal blockage, particularly in the morning, and sneezing after contact with house dust. Since the age of 10, the patient had received treatment for asthma. In infancy and up to the age of 2, he had been allergic to cow’s milk proteins, and a dairy-free diet was followed for that reason in this period. There were no cases of allergic diseases in the family history. The patient is a generally healthy person, who has received no chronic medical treatment apart from that which has been recommended for the allergy-related problems. He has a university-level educational background and works as an electrician. He lives in a large city, does not play sports or get involved in any hobbies. He does not have pets at home. The patient does not smoke cigarettes and occasionally consumes alcohol. Three years before, he had visited an allergologist at another clinical center due to the experienced complaints and was consequently diagnosed with allergic rhinitis and asthma. Skin prick tests (SPTs) were performed and, based on the results, the patient was qualified for specific immunotherapy involving the following vaccines: Allergovit (Allergopharma) 006 Grasses 100%, Allergovit (Allergopharma) 108 Birch 100%, and Novo-Helisen Depot (Allergopharma) 708 Dermatophagoides farinae 50% + 725 Dermatophagoides pteronyssinus 50%. During the course of treatment, the patient was showing good toleration and improvements, which consisted of the symptoms being reduced by about 30%. Because of his asthma, he was using a budesonide/formoterol combination spray, at a dose of one inhalation, two times a day; the disease was well-controlled as a result. In addition, due to his reported symptoms in the form of burning in the mouth, abdominal pain, hives, and facial erythema, following the ingestion of various foods, he had been prescribed epinephrine autoinjections. He was also taking bilastine, at a dose of 20 mg/d. About two years prior to presentation, the patient had also noticed a swallowing disorder in the form of the feeling of food being lodged in his throat, difficulty swallowing larger and harder bites of food, and the need to wash food down with copious amounts of water during all his meals. For this reason, he was referred to the Department of Gastroenterology, where eosinophilic esophagitis (EoE) was diagnosed, following a gastroscopic examination, with esophageal sampling. The patient was treated with a milk-, chicken egg-, wheat-, soy-, fish-, and seafood-exclusion diet, along with a proton pump inhibitor (PPI), namely pantoprazole, at a dose of 40 mg per day. Although minimal improvements were observed as a result, the patient did not agree to a follow-up gastroscopy after 8 weeks of treatment. A full physical examination was performed at our outpatient clinic, revealing no obvious pathology other than slight hypertrophy of the inferior turbinates. It turned out that the patient had completely abandoned the previously prescribed diet.

Due to the atypical course of the disease and the severity of the symptoms, an enzyme assay was performed, and the patient was found to present with a reduced DAO enzyme activity of 7.5 U/mL (reference range 14–33 U/mL), along with a normal tryptase level of 3.78 ng/mL (reference range below 10 ng/mL). The 50 min histamine skin test was not performed, due to the fact that the patient had been receiving antihistamines as part of a chronic regimen. A spirometry examination, with a reversibility test (FEV1%/VCmax 96%, SR-0.47, p 32 before; FEV1%/VCmax 113%, SR-0.11, p 44 after bronchodilator administration), revealed no signs of bronchial tree obstruction.

**Figure 1:**
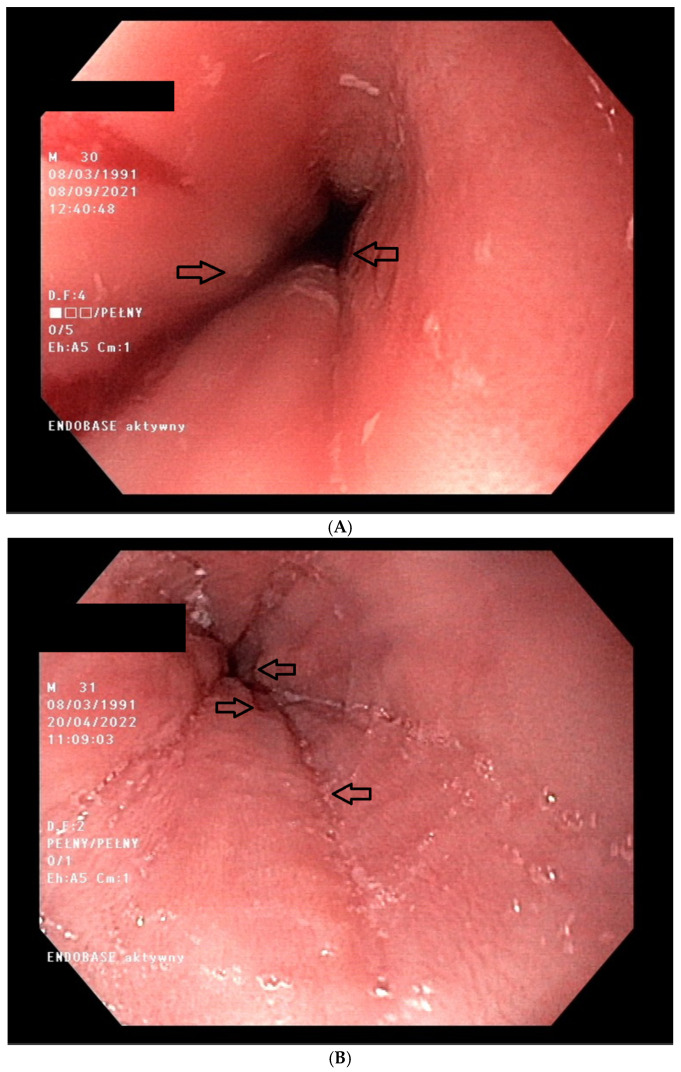
Image of the lower part of the patient’s esophagus. (**A**) The entire length of the esophagus is quite narrow, the mucous membrane is swollen and brittle, the walls are not susceptible to insufflation, the mucous membrane tears locally during insufflation, the lumen in the lower part of the esophagus is narrow but allows the endoscope to pass. The Z-line is irregular. (**B**) The esophagus has slow peristalsis; the mucous membrane is fragile, it bleeds easily when taking samples (also when the endoscope passes through); in the lower part of the esophagus, there are several areas of erosion, with a diameter of 3–4 mm; the Z-line irregular is irregular.

Due to the symptoms of eosinophilic esophagitis, the patient was referred to the Department of Gastroenterology, where an esophagogastroduodenoscopic examination was performed, along with esophageal specimen collection. The examination summary (camera type GIF–H190) was as follows: esophagus somewhat narrow along the entire length, peristalsis poorly expressed, mucosa slightly swollen, friable, locally rupturing upon insufflation; specimens were collected from the lower, middle, and upper part of the esophagus. The patient had an irregular Z-line, running along the upper border of the gastric mucosal fold (Figure 1A). The presentation of the gullet, fundus, body, and prepyloric sections of the stomach was unremarkable, as was that of the duodenal bulb and the initial section of the descending duodenum. The urease test was negative. The results of the histopathology examinations were summarized as follows: 1,2. unremarkable duodenal and gastric mucosa; 3,4. (lower and middle part of the esophagus) bands of squamous multilayered epithelium; eosinophils visible, 15/HPF, within the stroma, with the infiltration of mononuclear inflammatory cells being noted; 5. (upper part of the esophagus) mucosal fragments, hyperplasia of the basal layer, up to ½ of the epithelial thickness (“blue mucosa”), epithelial foci of up to 20 eosinophils/HPF, including surface layers; isolated eosinophilic microabscesses; single eosinophils can also be seen within the lining, with the infiltration of mononuclear inflammatory cells being noted.

In addition, a colonoscopic examination was performed at the Gastroenterology Department to rule out other inflammatory diseases of the gastrointestinal tract, yielding a negative result. Celiac disease and lactose intolerance were also ruled out. Based on the diagnostic workup, namely the esophagogastroduodenoscopy, colonoscopy, spirometry, plasma DAO enzyme activity analysis, and ruling out other differential diagnoses, like Celiac disease, lactose intolerance, and inflammatory bowel diseases (Crohn’s disease and ulcerative colitis), the patient was diagnosed with eosinophilic esophagitis and histamine intolerance.

## 4. Treatment and Follow-Up

The administered treatment included a diet with the exclusion of six food products, namely milk, eggs, soy, wheat, fish, and seafood, foods identified according to the Alex test results (Table 1), as well as cheese, alcohol, aged sausage, fermented ham, cured meat, spinach, eggplant, avocado, tomatoes, ketchup, chocolate, pickles, and yeast. The patient was referred to a nutritionist for the establishment of a complete diet. Budesonide, in the form of orally disintegrating tablets (Jorveza, Dr. Falk Pharma GmbH Leinenweberstr. 5 79108 Freiburg Germany), at a dose of 1 mg b.i.d., for 12 weeks, was also introduced. In addition, a diamine oxidase (DAO) preparation, Daosin (STADA Consumer Health Deutschland GmbH), was introduced, with the schedule of one tablet t.i.d. before main meals. The previous therapies for allergic rhinitis and asthma were left unchanged. The patient was thoroughly informed about the nature of his diseases, and the required treatment regimen was carefully communicated. At the follow-up examination, after one month of therapy, clinical improvements were observed. The patient reported no episodes of dysphagia and no need to use epinephrine autoinjectors. The symptoms of histamine intolerance and food allergies were almost completely resolved. However, the patient could not fully tolerate the established diet and complained about the significant restrictions in regard to eating his favorite foods. At the time of the follow-up examination, 12 weeks after the implementation of the therapy, the patient again complained about experiencing difficulty swallowing, a burning sensation upon swallowing, and the need to wash food down with large amounts of water. It was established that the patient had not adhered to the proposed diet, nor had he taken his medication regularly; moreover, he had previously consumed alcohol, at least once a week. An esophagogastroduodenoscopy was performed again at the Department of Gastroenterology. The examination summary (camera type GIF-H190, Olympus Deutschland GmbH, Amsinckstraße 6320097, Hamburg, Germany), was as follows: esophageal peristalsis impaired, mucosa friable, bleeds easily upon collection of the specimens and even passing the endoscope; the specimens were collected from the lower, middle, and upper part of the esophagus. Several areas of erosion, 3–4 mm in diameter, were found within the lower part of the esophagus. The Z-line was irregular, running along the upper border of the gastric mucosal fold (Figure 1B). The gullet, fundus, body, and pyloric sections of the stomach were unremarkable. The duodenal bulb and the initial section of the descending duodenum were unremarkable; sections were collected.

**Figure 2:**
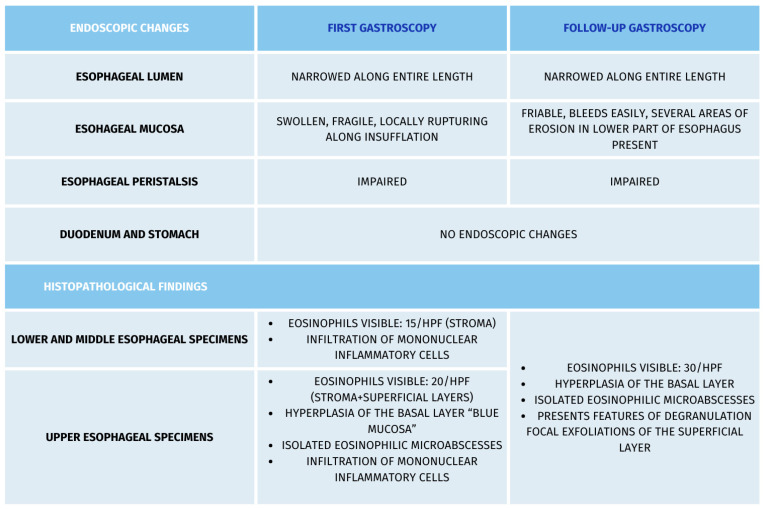
The results of the esophagogastroduodenoscopic and histopathology examinations.

The results of the histopathology examinations were summarized as follows: all esophageal specimens presenting with mucosal fragments, covered by multilayered squamous epithelium, with focal exfoliations of the superficial layer, hyperplasia of the basal layer, epithelial foci of up to 30 eosinophils/HPF, some of them with features of degranulation, isolated eosinophilic microabscesses. Sections from the stomach and duodenum presented with unremarkable mucosal membranes (Figure 2).

**Figure 3:**
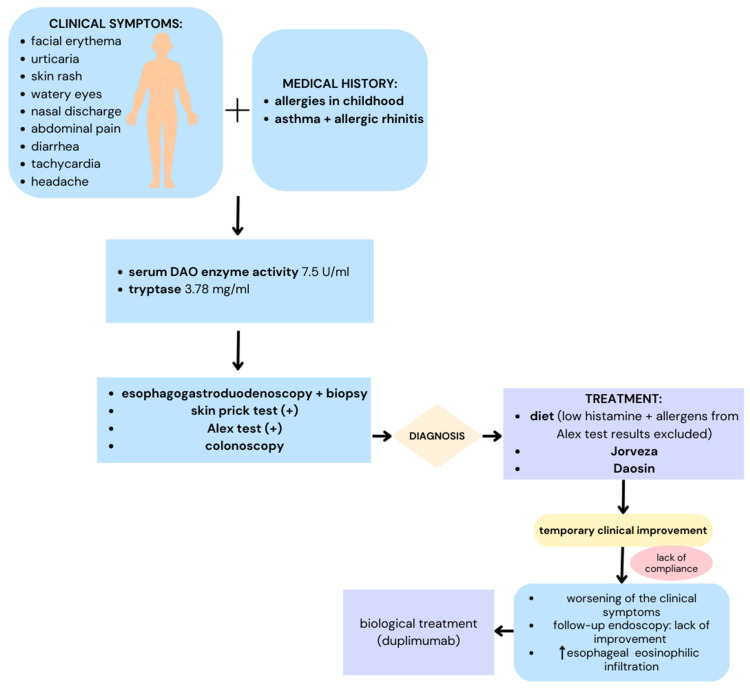
A schematic summary of the case presented in this paper, with an overview of the medical history, diagnostic and treatment workflow, and follow-up.

As the clinical and histopathological presentation of eosinophilic esophagitis worsened, the patient was referred to another clinical center to be qualified for biological treatment (Dupilumab). DAO enzyme supplementation and the existing treatment were maintained (Figure 3).

## 5. Literature Review and Discussion

In this paper, we are presenting a very interesting and rare case involving the coexistence of histamine intolerance with polyvalent allergy, in a patient with allergic multimorbidity (allergic rhinitis, asthma, food allergies, and eosinophilic esophagitis). In recent years, the problem of the co-occurrence of allergic and non-allergic diseases has been widely addressed in the literature. In 2015, Bousqet et al. reported on the phenomenon of allergic multimorbidity, including allergic rhinitis, asthma, and atopic dermatitis, noting the similar immunological and non-immunological pathomechanisms of these conditions [11]. Cingi et al. reported on the phenomenon of multimorbidity in relation to allergic rhinitis. The authors proposed that the diseases coexisting with allergic rhinitis be classified into: (a) allergic diseases (asthma, atopic dermatitis, food allergies, anaphylaxis); (b) diseases located in anatomical proximity to the nose (otitis media, sinusitis, conjunctivitis, eosinophilic esophagitis), (c) sleep and concentration problems; and (d) nasal turbinate hypertrophy [12]. In regard to the patient described in this paper, the rapid onset of symptoms and the atypical course of the disease made it necessary to expand the diagnostic tests in order to diagnose a DAO enzyme deficiency.

Over the past two decades, the number of food-related adverse reactions has increased significantly. Up to 20–30% of the Western population has reported symptoms following the ingestion of foods; however, food allergies and intolerances have been well-documented in only 3.6% of the population [13]. At present, the prevalence of food allergies is estimated to be 6–10% in the pediatric population and 2–5% in adults [14]. As of today, it is difficult to precisely determine the prevalence of histamine intolerance, but it is estimated to be around 1%, while reduced DAO activity can occur in up to 15% of the population. Traditionally, the diagnosis of histamine intolerance was established on the basis of sensitization being ruled out based upon the presence of allergy symptoms, as well as the improvement of symptoms following introduction of a low-histamine diet. Recent studies have shown that the determination of serum DAO activity was most helpful in the diagnosis of histamine intolerance [15,16,17,18,19]. Pinzer et al. evaluated daily fluctuations in histamine levels and DAO activity in three groups of patients: those with histamine intolerance, those with confirmed food allergies, and healthy subjects. Twenty-four percent of the patients (8 out of 33) presented with elevated histamine levels, probably due to a significant reduction in the daytime DAO activity compared to the other patients. The authors showed that the measurement of histamine levels and DAO activity during the daytime was very useful in regard to the differential diagnosis of histamine intolerance, as it was more precise and showed that there was no correlation between the serological parameters and the patients’ complaints [16].

The co-occurrence of allergies and histamine intolerance (overlap syndrome) significantly increases the severity of the course of allergic diseases. In his work, Buczyłko described nine clinical cases of overlap syndrome between histamine intolerance and other allergic diseases (rhinitis, urticaria, allergic dermatitis, or food allergies). In all cases, the correct diagnosis of histamine intolerance and appropriate management had led to therapeutic success and significantly increased the quality of the patients’ lives [15]. Mayo-Yáñez et al. evaluated the prevalence of DAO enzyme deficiency in patients with idiopathic rhinitis, finding that among 116 patients with the condition, DAO deficiency was present in 41.38%. Moreover, the authors showed that in patients with mild idiopathic rhinitis, the DAO enzyme activity was significantly reduced, as compared to patients with moderate and severe rhinitis, and the reduction in DAO activity was inversely proportional to the degree of nasal obstruction, as assessed by the PNIF method [17].

A novel direction in diagnostics consists of the identification of selected SNPs (i.e., rs10156191, rs1049742, rs1049793) of the AOC1 gene that are associated with reduced DAO activity. Notably, the test reflects only a genetic predisposition to DAO deficiency; however, it is well-known that histamine intolerance frequently develops secondarily to gastrointestinal pathologies that result in reduced DAO levels. This diagnostic approach can be considered in cases involving symptoms characteristic of histamine intolerance that are of a heritable nature [3]. The current management of histamine intolerance includes the use of a histamine-reduced diet, supplementation with exogenous DAO, and the use of antihistamines. This management protocol was also followed in regard to the presented case, with the patient showing improvements as a result of the treatment. The diagnosis of histamine intolerance is difficult, due to the high variability and heterogeneity of the clinical symptoms in individual patients. Many studies on the issue recommend ruling out an allergic background in terms of the complaint. However, the possibility of the symptoms of an IgE-dependent allergy overlapping with those of histamine intolerance should be taken into account in every case. This is particularly important in patients presenting with an atypical and severe course of allergic diseases [18,19,20].

Our patient was found to be allergic to multiple allergens and multimorbid in relation to allergic diseases; in the case of histamine intolerance, this further significantly hinders a proper diagnosis and affects the strategies for proper treatment. Histamine intolerance is of increasing interest to both researchers and clinicians. The disorder affects adults, as well as children. The current knowledge and research have clarified many of the previous uncertainties, but generally accepted diagnostic and treatment standards are still lacking [20]. It should be noted that the data on DAO concentrations in patients with HIT are not consistent, with some authors not having observed a significant reduction in DAO levels in this group [3,20]. In addition, not all cases of HIT demonstrate marked improvements following the introduction of a low-histamine diet [21]. In their study, Bent et al. used a low-histamine diet in patients with suspected histamine intolerance, measured the serum DAO levels, and carried out a single-blind, placebo-controlled histamine challenge test to confirm histamine intolerance. The authors concluded that the histamine challenge test is a reliable and sensitive diagnostic tool, while diet adherence and serum DAO determinations are characterized by low specificity when used as markers of histamine intolerance [21]. However, it should be noted that the histamine challenge test involves oral administration of the substance and requires medical supervision and hospitalization of the patient. The use of this method is limited due to the risk of serious adverse effects and the lack of a standardized histamine dose or a properly established protocol [20]. An interesting study was conducted by Shi et al., who demonstrated that high serum DAO concentrations significantly alter the intestinal microflora, thus leading to gastrointestinal complaints. On the other hand, the authors found no relationship between serum cytokine and DAO levels [22].

In 2024, an intriguing retrospective study by Duelo et al. was published, in which the authors investigated the frequency of four variants of the AOC1 gene, encoding diamine oxidase (DAO), in Caucasian adults experiencing histamine intolerance. Within a sample comprising 100 patients and 100 healthy controls, non-synonymous single nucleotide variations (SNVs) in the DAO gene were identified through multiplex single-nucleotide primer extension (SNPE) and capillary electrophoresis [23]. The serum DAO activity was assessed using a radio-extraction assay. The findings revealed that 79% of participants presenting with histamine intolerance symptoms possessed at least one of the four SNVs linked to diminished DAO activity. Additionally, a marginally greater, yet statistically significant, proportion of patients exhibited elevated genetic risk scores, indicative of the combined influence of carrying multiple DAO deficiency-related gene variants and a substantial burden of risk alleles (homozygous). A notable association emerged between serum DAO activity and the genetic burden of a particular SNV, with DAO activity markedly lower in individuals homozygous for rs2052129. These findings suggest that the aggregation of multiple DAO deficiency-related gene variants and a pronounced load of risk alleles (homozygous) holds greater significance than the existence of individual SNVs alone. Further investigations are required to evaluate the predictive capacity of these specific DAO gene variants. By mediating interactions among mast cells, microglia, and astrocytes, histamine plays a crucial role in neuroinflammation. This makes histamine a promising therapeutic target, with potential to prevent or manage the symptoms of various neurodevelopmental disorders (NDDs) [23,24]. The association between DAO dysfunction and neurodegenerative diseases is also well-documented. Dave et al., in their next-generation sequencing study, identified the E121K mutation in the DAO gene, which has been linked to amyotrophic lateral sclerosis (ALS) in patients from India [25].

The immune system is sensitive to histamine levels, and disturbances caused by DAO mutations can manifest as altered immune cell activity and an altered cytokine profile. Elevated histamine levels disrupt the immune response. The activation of mast cells through H4R not only triggers the production of proinflammatory cytokines and chemokines, including IL-6, TNF-α, TGF-β1, RANTES, IL-8, MIP-1α, and MCP-1, but also enhances mast cell migration to allergic reaction sites. Additionally, this stimulation elevates FcεRI expression and its surface presentation on mast cells, facilitating degranulation by increasing intracellular calcium mobilization [26,27,28].

## 6. Conclusions

Histamine intolerance is a condition caused by an imbalance between the amount of histamine present in the body and the body’s ability to break it down. Histamine intolerance most often coexists with a deficiency in terms of the DAO enzyme, which breaks down histamine in food. Establishing the diagnosis of HIT is difficult due to the fact that individual patients may present with widely variable and diverse symptoms, simultaneously involving multiple organs in the body. Diagnosis requires a complex, time-consuming, and multidisciplinary approach, including the differentiation of disorders with similar manifestations. The diagnosis of histamine intolerance is possible after the exclusion of an allergic background to the complaints, with a note being taken, however, of the possible overlap between an IgE-mediated allergy and histamine intolerance, as was observed in the presented case. The presented clinical case may be helpful in the daily practice of allergists and physicians with other specialties, as an example of multimorbidity with both allergic and non-allergic origins.

## Figures and Tables

**Table 1:** Assessment of sensitization components (Alex test).

Grass pollen	
Bermuda grass	Cyn d 1, beta-expansin—3.62 kUA/L
Timothy grass	Phl p 1, beta-expressin—9.15 kUA/L; Phl p 2, expansin—6.41 kUA/L
Rye pollen	Sec c_pollen—1.24 kUA/L
**Tree pollen**	
Silver birch	Bet v 1, PR-10—42.25 kUA/L; Bet v 2, prophyllin—25.16 kUA/L; Bet v 6, isoflavone reductase—32.52 kUA/L
Beech	Fag s 1, PR-10—27.18 kUA/L
**House dust mites**	
Dermatophagoides farinae	Der f 2, NPC2 family –20.99 kUA/L
Dermatophagoides pteronyssinus	Der p 1, Cysteine protease—1.47 kUA/L, Der p 2, NPC2 family—28.51 kUA/L, Der p 23—Peritrophin-like domain protein—5.84 kUA/L
**Molds**	
Malassezia sympodialis	Mala s 11, Mitochondrial superoxide dismutase—3.11 kUA/L
Alternaria alternata	Alt a 1, Group Alt a 1—20.87 kUA/L
**Legumes**	
Peanut	Ara h 8—PR-10—26.93 kUA/L
Soybeans	Gly m 4—PR-10—14.87 kUA/L
**Fruits**	
Strawberry	Fra a 1+3, PR 10+LTP—9.68 kUA/L
Apple	Mal d 1, PR-10—9.84 kUA/L
**Vegetables**	
Celery	Api g 1, PR-10—17.82 kUA/L
Carrots	Dau c 1, PR-10—23.65 kUA/L
**Nuts**	
Hazelnut	Cor a 1.0401, PR-10—26.57 kUA/L
**Animal allergens**	
Dog	Can f 1, Lipocalin—2.27 kUA/L
Cat	Fel d 1, Secretoglobin—5.56 kUA/L

## Data Availability

The data presented in this study are available on request from the corresponding author.

## Abbreviations

HIT: histamine intolerance; DAO: diamine oxidase; SNP: single nucleotide polymorphism; SPTs: skin prick tests; EoE: eosinophilic esophagitis.

## References

[1] Comas-Basté O., Sánchez-Pérez S., Veciana-Nogués M.T., Latorre-Moratalla M., Vidal-Carou M.d.C. (2020). Histamine Intolerance: The Current State of the Art. Biomolecules.

[2] Reese I., Ballmer-Weber B., Beyer K., Fuchs T., Kleine-Tebbe J., Klimek L., Lepp U., Niggemann B., Saloga J., Schäfer C. (2017). German guideline for the management of adverse reactions to ingested histamine: Guideline of the German Society for Allergology and Clinical Immunology (DGAKI), the German Society for Pediatric Allergology and Environmental Medicine (GPA), the German Association of Allergologists (AeDA), and the Swiss Society for Allergology and Immunology (SGAI). Allergo J. Int..

[3] Reese I., Ballmer-Weber B., Beyer K., Dölle-Bierke S., Kleine-Tebbe J., Klimek L., Lämmel S., Lepp U., Saloga J., Schäfer C. (2021). Guideline on management of suspected adverse reactions to ingested histamine: Guideline of the German Society for Allergology and Clinical Immunology (DGAKI), the Society for Pediatric Allergology and Environmental Medicine (GPA), the Medical Association of German Allergologists (AeDA) as well as the Swiss Society for Allergology and Immunology (SGAI) and the Austrian Society for Allergology and Immunology (ÖGAI). Allergol Select..

[4] EFSA Panel on Biological Hazards (BIOHAZ) (2011). Scientific Opinion on risk based control of biogenic amine formation in fermented foods. EFSA J..

[5] Legroux R., Bovet D., Levaditi J.C. (1947). Présence d’histamine dans la chair d’un thon responsable d’une intoxication collective [Presence of histamine in the flesh of a tuna responsible for collective poisoning]. Ann. Inst. Pasteur.

[6] Hungerford J.M. (2021). Histamine and Scombrotoxins. Toxicon.

[7] Taylor S.L., Eitenmiller R.R. (1986). Histamine food poisoning: Toxicology and clinical aspects. CRC Crit. Rev. Toxicol..

[8] Zhao Y., Zhang X., Jin H., Chen L., Ji J., Zhang Z. (2022). Histamine Intolerance—A Kind of Pseudoallergic Reaction. Biomolecules.

[9] Best C.H. (1929). The disappearance of histamine from autolysing lung tissue. J. Physiol..

[10] Hrubisko M., Danis R., Huorka M., Wawruch M. (2021). Histamine Intolerance-The More We Know the Less We Know: A Review. Nutrients.

[11] Bousquet J., Anto J.M., Wickman M., Keil T., Valenta R., Haahtela T., Lodrup Carlsen K., van Hage M., Akdis C., Bachert C. (2015). Are allergic multimorbidities and IgE polysensitization associated with the persistence or re-occurrence of foetal type 2 signalling? The MeDALL hypothesis. Allergy.

[12] Cingi C., Gevaert R., Mosges R., Rondon C., Hox V., Rudenko M., Muluk N.B., Scadding G., Manole F., Hupin C. (2017). Multi-morbidities of allergic rhinitis in adults: Eu-ropean Academy of Allergy and Clinical Immunology Task Force Report. Clin. Trans. Allergy.

[13] Zingone F., Bertin L., Maniero D., Palo M., Lorenzon G., Barberio B., Ciacci C., Savarino E.V. (2023). Myths and Facts about Food Intolerance: A Narrative Review. Nutrients.

[14] Sicherer S.H., Sampson H.A. (2018). Food allergy: A review and update on epidemiology, pathogenesis, diagnosis, prevention, and management. J. Allergy Clin. Immunol..

[15] Buczyłko K. (2022). Usefulness of DAO biomarker in difficult allergy: Consideration based on own typical cases. Alergol. Pol-Ska Pol. J. Allergol..

[16] Pinzer T.C., Tietz E., Waldmann E., Schink M., Neurath M.F., Zopf Y. (2017). Circadian profiling reveals higher histamine plasma levels and lower diamine oxidase serum activities in 24% of patients with suspected histamine intolerance compared to food allergy and controls. Allergy.

[17] Mayo-Yáñez M., Díaz-Díaz A., Calvo-Henríquez C., Lechien J.R., Vaira L.A., Figueroa A. (2023). Diamine Oxidase Activity Deficit and Idiopathic Rhinitis: A New Subgroup of Non-Allergic Rhinitis?. Life.

[18] Reese I., Zuberbier T., Bunselmeyer B., Erdmann S., Henzgen M., Fuchs T., Jäger L., Kleine-Tebbe J., Lepp U., Niggemann B. (2009). Diagnostic approach for suspected pseudoallergic reaction to food ingredients. J. Dtsch. Dermatol. Ges..

[19] Son J.H., Chung B.Y., Kim H.O., Park C.W. (2018). A Histamine-Free Diet Is Helpful for Treatment of Adult Patients with Chronic Spontaneous Urticaria. Ann. Dermatol..

[20] Buczyłko K., Bartnicka A., Kruszewski J., Plata-Nazar K., Piwowarek K., Kupczyk M., Gawlik R., Lebensztejn D., Bartuzi Z., Mazela J. (2023). Guidelines for the diagnosis and management of histamine intolerance. Pol. J. Allergol..

[21] Bent R.K., Kugler C., Faihs V., Darsow U., Biedermann T., Brockow K. (2023). Placebo-Controlled Histamine Challenge Disproves Suspicion of Histamine Intolerance. J. Allergy Clin. Immunol. Pr..

[22] Shi L.M., Li Y., Liu Y.B., Jia H.M. (2022). Alterations of gut microbiota and cytokines in elevated serum diamine oxidase disorder. Medicine.

[23] Duelo A., Comas-Basté O., Sánchez-Pérez S., Veciana-Nogués M.T., Ruiz-Casares E., Vidal-Carou M.C., Latorre-Moratalla M.L. (2024). Pilot Study on the Prevalence of Diamine Oxidase Gene Variants in Patients with Symptoms of Histamine Intolerance. Nutrients.

[24] Carthy E., Ellender T. (2021). Histamine, Neuroinflammation and Neurodevelopment: A Review. Front. Neurosci..

[25] Dave U., Khan S., Gomes J. (2023). Characterization of E121K mutation of D-amino acid oxidase—Insights into mechanisms leading to amyotrophic lateral sclerosis. Biochim. Biophys. Acta Proteins Proteom..

[26] Thangam E.B., Jemima E.A., Singh H., Baig M.S., Khan M., Mathias C.B., Church M.K., Saluja R. (2018). The Role of Histamine and Histamine Receptors in Mast Cell-Mediated Allergy and Inflammation: The Hunt for New Therapeutic Targets. Front. Immunol..

[27] Shulpekova Y.O., Nechaev V.M., Popova I.R., Deeva T.A., Kopylov A.T., Malsagova K.A., Kaysheva A.L., Ivashkin V.T. (2021). Food Intolerance: The Role of Histamine. Nutrients.

[28] Jochum C. (2024). Histamine Intolerance: Symptoms, Diagnosis, and Beyond. Nutrients.

